# A comparison of selenium concentrations in selected organs of wild boar (*Sus scrofa*) from industrialized and non-industrialized regions of Poland

**DOI:** 10.1007/s11356-018-1263-4

**Published:** 2018-01-23

**Authors:** Ewa Nowakowska, Bogumiła Pilarczyk, Renata Pilarczyk, Agnieszka Tomza-Marciniak, Małgorzata Bąkowska

**Affiliations:** 1 Department of Animal Reproduction Biotechnology and Environmental Hygiene, West Pomeranian University of Technology in Szczecin, Klemensa Janickiego 29, 71-270 Szczecin, Poland; 2 Laboratory of Biostatistics, West Pomeranian University of Technology in Szczecin, Klemensa Janickiego 29, 71-270 Szczecin, Poland

**Keywords:** Selenium, Liver, Kidney, Industrialized area, Wild boar

## Abstract

The aim of this study was to compare selenium concentration in the liver and kidneys of wild boar inhabiting industrialized and non-industrialized regions of Poland. Selenium concentrations in organs were determined using spectrofluorometric method. In all the animals studied, Se concentrations were a few times lower than in kidneys which may indicate too low content of this element either in the boar’s diet or the presence of a poorly absorbable form of Se. No statistically significant differences were noted in the mean Se concentrations in the liver and kidney of wild boar from industrialized and non-industrialized areas. In the case of wild boar, it seems that the level of selenium in their organs is more dependent on geochemical conditions in the specific feeding ground than on the scale of regional industrialization, and that this situation is most likely related to the specificity of wild boar feeding.

## Introduction

The concentration of selenium in the tissues of free-living animals depends on type of consumed food, and directly or indirectly, on the content of selenium in the environment, especially in the soil (Flueck et al. 2012; Pilarczyk et al. 2009; Sablik et al. 2011; Seremak et al. 2011). Therefore, thanks to a complete and lifelong integration with the environment, free-living animals are found to be good indicators of content of selenium in the environment. Free-living species, in contrast to farm animals whose food compositions are not that strongly related to local environmental conditions, are suited to be used as bioindicators of Se content in a particular area.

Studies on the content of selenium in the environment include among other things measurements of its concentration in the organs of free-living animals related to land ecosystems. Many different species like wild boar (*Sus scrofa*), red deer (*Cervus elaphus*), and fallow deer (*Dama dama*) have been used in such studies (Amici et al. 2012; Humann-Ziehank et al. 2008; Lazarus et al. 2008; Pilarczyk et al., 2010; Vikøren et al. 2005; Vikøren et al. 2011).

Selenium is one of the most active bioelements. As a component of selenoproteins, it plays both a structural and an enzymatic role. The most important selenoproteins include glutathione peroxidase, iodothyronine deiodinase, P and W selenoproteins, and thioredoxin reductase (Flohe et al. 2000; Gladyshev 2001; Rayman 2000). The liver plays a most important role in maintaining the selenium homeostasis in an organism, as its cells are responsible for the synthesis and distribution of selenoproteins (including Se-dependent glutathione peroxidase) (Czuczejko et al. 2003).

Although clinical symptoms of selenium deficiencies are rarely observed in free-living species, there still is a risk of occurrence of subclinical symptoms that are not obvious to woodsmen, veterinarians, and huntsmen. In free-living species like roe deer, red deer, and fallow deer, a white muscle disease may be observed, and as it contributes to a decreased ability to escape from predators, such situation may cause a decrease in the population size of these animals in the environment (Hnilicka et al. 2004). In females, as a result of a selenium deficiency, milk production may decrease and lead to a quicker weaning of infants, causing them to become an easier prey for predators. A lowered supply in Se in the diet may also cause hind infertility and births of weak calves. In males, due to a lowered supply of Se, reproductive potential may decrease and cause disturbances in the gender and age structure of a herd, and a worsened quality of individuals as well. A deficiency of selenium in free-living animals also results in disturbances in bone mineralization processes, joint degeneration, reduced bone density, and induction of periodontal diseases (Flueck et al. 2012; Flueck and Smith-Flueck 2008; Hnilicka et al. 2004).

A prolonged deficiency of selenium in free-living species can affect the number of offspring, general condition of animals, embryo decay, placental retention, growth rate, embryonal mortality, and age of onset of puberty (Flueck et al. 2012; Flueck and Smith-Flueck 2008). One of the main reasons of a decreased population size in some species of free-living animals—apart from such factors as irreversible changes in animal habitats, large-scale agriculture, changes in the structure of farmed crops and predation—may be also the reduced disease resistance of animals caused by a deficiency in selenium supply (Flueck et al. 2012).

The aim of this study was to compare the content of selenium in the liver and kidneys of wild boar inhabiting industrialized and non-industrialized regions of Poland.

## Material and methods

### Samples

**Fig. 1:**
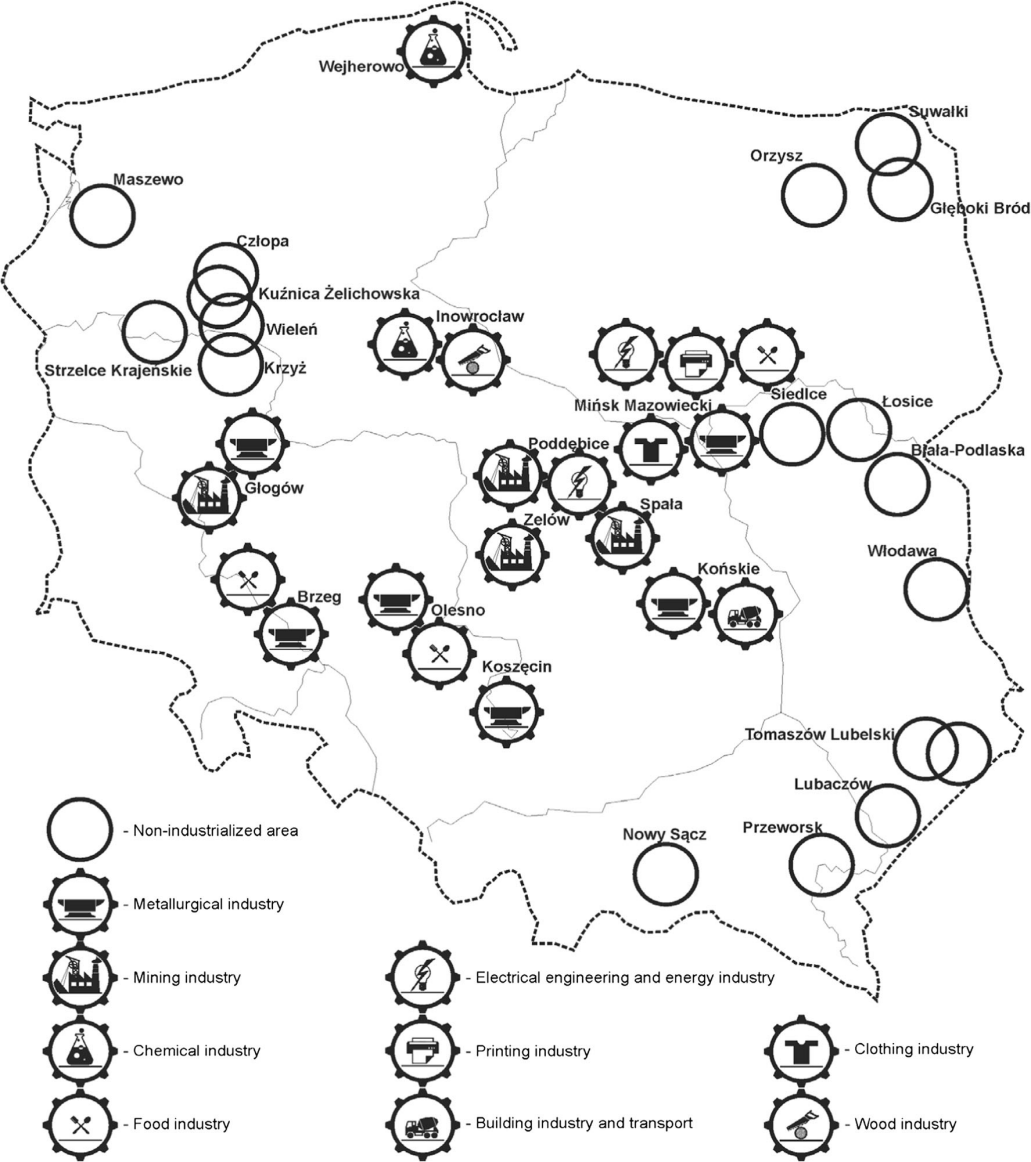
The regions of sample collection in relation to the area industrialization

The research material included samples of liver and kidneys of wild boar (*n* = 175, aged 2–4 years) hunted between 15th August and 15th January 2010. The animals were provided by qualified huntsmen in 28 game hunting districts located in all 16 voivodeships of Poland during the hunting season and without exceeding the legally accepted hunting limits. The hunting districts where the samples were collected did not use selenium supplemented salt blocks. The dates of hunting were chosen in agreement with the directive of Ministry of Environment (16th March 2005) about the permissible game animal hunting seasons (Dz. U. of 25th March 2005). The level of industrialization in the regions of sample collection are shown in Fig. 1.

The huntsmen evaluated the age of the animals based on the guidebook by Przybylski et al. (2010).

The research material (liver and kidneys) was collected and frozen at − 20 °C until the time of laboratory analyzes.

### Chemical analyses

The concentration of selenium was measured according to the Watkinson method, modified by Grzebuła and Witkowski (1977). The samples of liver (~ 1 g) and kidney (~ 0.5 g) were digested in HNO_3_ at 230 °C for 180 min and in HClO_4_ at 310 °C for 20 min. Then, 9% HCl was added to the mineralized samples to reduce selenates (Se VI) to selenites (Se IV). Selenium was derivatized with 2,3-diaminonaphthalene (DAN) under controlled pH (pH 1–2) until the formation of selenodiazole complex. This complex was extracted into cyclohexane. Se concentration was determined fluorometrically using a Shimadzu RF-5001 PC spectrofluorophotometer. Fluorescence was measured using an emission wavelength of 518 nm and an excitation wavelength of 378 nm. Concentration of selenium was calculated into 1 g of wet and dry matter. Dry matter was measured for each sample.

The accuracy of the analytical procedure was verified by measuring the level of selenium in a reference material: NCS ZC 71001 (Beef Liver) (China National Analysis Center for Iron and Steel Beijing China). Mean recovery was 91.1% of the reference value.

### Dry matter determination

One gram samples of fresh liver and kidney were placed into clean and dry weighing bottles, then dried at 105 °C to a constant weight, until the absolute difference in dry matter content between two subsequent measurement did not differ more than 0.1%.

The concentration of selenium was calculated into 1 g of wet and dry matter.

### Statistical analysis

The results were analyzed statistically using STATISTICA 9.0 PL software. The tables of results include arithmetic means, median, standard error of measurement (SEM), and minimal and maximal values (range). An evaluation of a normal distribution in the examined variables was performed with a Shapiro-Wilk test, and to ensure a normal distribution, a logarithm of the variables was calculated. Statistical analysis was performed on the logarithms of values obtained in the study. One-way ANOVA was used to examine the effect of each factor separately, and the significance of differences between the mean values was calculated with a Duncan test. The interdependence between the concentration of selenium in the liver and kidneys of the examined animals was determined with a Pearson (*r*_x,y_) correlation coefficient.

## Results

The mean concentrations of selenium in the liver of wild boar from the non-industrialized (0.232 μg/g w.w.; 0.790 μg/g d.w.) and industrialized (0.227 μg/g w.w.; 0.776 μg/g d.w.) areas were similar, with marginally higher values observed in the animals from non-industrialized regions (Table 1). No statistically significant differences were noted in the mean concentrations of selenium in the liver of wild boar with respect to the region of sample origin (industrialized, non-industrialized).

**Table 1:** The mean concentration of selenium in livers and kidneys of examined wild boars from industrialized and non-industrialized regions

Region	*n*	Concentration of Se (μg/g w.w.)			
		Mean	Median	SEM	Range
Liver					
Non-industrialized	94	0.232	0.207	0.012	0.056–0.626
Industrialized	81	0.227	0.204	0.013	0.036–0.554
Kidney					
Non-industrialized	94	1.303	1.224	0.052	0.415–2.679
Industrialized	81	1.355	1.167	0.085	0.322–4.286

The mean concentrations of selenium in the kidney of wild boar from the non-industrialized (1.303 μg/g w.w.; 4.446 μg/g d.w.) and industrialized (1.355 μg/g w.w.; 4.623 μg/g d.w.) regions were also similar, with no statistically significant differences noted between the regions of sample origin (industrialized, non-industrialized).

A significant positive correlation (*p* ≤ 0.001) was found between the concentrations of selenium observed between the liver and kidney (*r*_x,y_ = 0.67 and *r*_x,y_ = 0.70, respectively) in the wild boar from both the industrialized and non-industrialized areas (Table 2).

**Table 2:** A correlation between the levels of selenium in kidney and liver of wild boars from industrialized and non-industrialized regions

Region	n	Correlation coefficient (*r*_x,y_)
Non-industrialized	94	0.67***
Industrialized	81	0.70***
Total	175	0.67***

Correlation coefficient significant at: *** *P* ≤ 0.001

## Discussion

Chemical elements that are released into the environment as a result of different industrial processes can be a very important factor that affects Se bioavailability in the early stages of the trophic chain, which in turn indirectly affects the concentration of this element in animal organisms. This situation is caused by the fact that elements belonging mostly to the group of heavy metals are strong selenium antagonists, and their presence in large amounts may lead ultimately to a reduce in the absorption of Se in an organism (He et al. 2004). Apart from Se antagonists, an acidic pH and increased concentrations of sulphur and phosphorus are also important factors that limit Se absorbance in the environment (Gupta and Watkinson 1985, Johnson 1991).

Selenium absorption in animals depends not only on the chemical composition of the food but also on the interactions between Se and other elements. As reported by Floriańczyk (1999), an antagonistic relationship occurs between Se and Cd and Pb. The presence of either of these two metals reduces the absorption of Se from the diet.

Based on this data, we assumed that the animals feeding in the industrialized areas would present lower Se concentrations in their tissues, respective of the lower absorption of this element from the environment (soil-plants-animal). However, in our study no such significant differences were noted in the mean concentration of selenium in the organs of the wild boar between the industrialized and non-industrialized areas. Interestingly, Reglero et al. (2009a) also observed that wild boar from a mining area had less Se in the liver than those from the control sites (0.453 vs. 0.589 μg/g dry weight). At the same time these authors had noted an opposite situation in red deer. This means that the method of food intake and the type of food play an important role in the intake of the necessary elements and in exposure to environmental pollutants as well. Wild boar, opposite to ruminants, is an omnivorous animal which can consume all available food sources (Herrero et al. 2006). More than 90% of their diet is plants and about 10% animals. While searching for food, wild boars root (dig) the soil, usually in a large area and relatively deep. The diet of wild boars includes earthworms, insects and their larvae, small rodents, eggs and chicks of ground-nesting birds, frogs, juvenile individuals of some mammal species, and carrion (Fronseca 2008; Massei and Genov 2004; Schley and Roper 2003).

In this study, we have shown a positive and significant correlation between the concentration of Se in wild boar liver and kidneys obtained in both regions. Accordingly, Lopez Alonso et al. (2004) have noted some significant associations between hepatic and renal concentration of Se and also Co and Cd. Concentration of Se in liver reflects a current supply in this element. In turn an increase of Se concentration in kidneys results from the fact that Se forms complexes with heavy metals—selenides. These complexes accumulate in kidneys and hereby metals are excluded from biochemical processes (Chavez 1981, Orłowski 2008).

The liver is the main organ responsible for selenium homeostasis in these animals. Selenium deficiencies result in emptying the reserves of this microelement, which is deposited mostly in liver. Thus, in the case of a selenium deficiency, the most pronounced changes in selenium levels are observed in this organ. For this reason, liver are a better indicator of selenium status than those in kidneys (Pollock 2005).

Oh et al. (1976) indicate the mutual proportions between the content of selenium in the liver and kidneys. As reported by many authors, animals fed with Se deficient food have always presented a higher concentration of Se in the kidneys than in the liver. A different situation was observed in animals fed with Se abundant fodder where the concentration of selenium was higher in the liver than the kidneys of the animals. In our study, we have shown that mean Se concentration in the wild boar liver was several times lower than in the kidneys (0.230 vs 1.327 μg/g w.w.), which may be evidence either of an insufficient content of this element in the diet or the presence of a poorly absorbable form of Se. It has to be mentioned that in wild boar, an evaluation of Se status is complicated due to the lack of reference values. However, compared with reference values presented for swine, the levels of selenium observed in our study can be considered marginal.

According to Puls (1994), the biochemical criteria used in evaluation of Se status for swine are (a) liver: below 0.11 μg/g m.m.—deficiency; 0.12 to 0.39 μg/g m.m.—marginal level; above 0.40 μg/g m.m.—optimal level for animals and (b) kidneys: below 0.77 μg/g m.m.—deficiency; 0.78 to 1.49 μg/g m.m.—marginal level; above 1.50 μg/g m.m.—optimal level for animals. The mean selenium concentrations observed in this study in the liver and kidneys are comparable to the results of earlier studies in the same area of Poland, considered as selenium-deficient (Pilarczyk et al. 2010).

Improper selenium status in free-living animals, like wild boar, may have severe implications, as Se deficiency is related to such disorders as white muscle disease, mulberry heart disease, and hepatosis diaetetica. In females, it can lead to the death of fetuses due to arrested development, retained placenta after delivery, inflammation of the genital tract and MMA syndrome (Grela and Sembratowicz 1997; Radostits et al., 2000). As the quality of semen is also strictly related to the concentration of selenium in the testes, a decreased level of this element may lead to a reduction in male reproductive potential (Reglero et al. 2009b).

## Summary

No reference values that relate to selenium concentrations in the organs of wild boar preclude a definitive evaluation of selenium status in this species. However, based on the proportions between concentrations of selenium in the liver and kidneys, we may assume that a Se deficiency was present in the animals tested, related either to a too low content of this element in their diet or to the presence of Se in a poorly absorbable form. It seems that the level of selenium in the organs of wild boar is more dependent on geochemical conditions in the specific feeding area than on the scale of regional industrialization, and this situation is most likely related to the specificity of wild boar feeding.
